# West Nile Virus: Epidemiology, Surveillance, and Prophylaxis with a Comparative Insight from Italy and Iran

**DOI:** 10.3390/vaccines14010057

**Published:** 2026-01-03

**Authors:** Soroosh Najafi, Maryam Jojani, Kianoosh Najafi, Vincenzo Costanzo, Caterina Vicidomini, Giovanni N. Roviello

**Affiliations:** 1 Scientific and Production Center “Armbiotechnology”, NAS RA, 14 Gyurjyan Str., Yerevan 0056, Armenia; 2 School of Medicine and Surgery, University of Naples ‘Federico II’, Via S. Pansini 5, 80131 Naples, Italy; 3 Institute of Molecular Biology and Pathology (IBPM), CNR National Research Council of Italy, 00185 Rome, Italy; 4 Institute of Biostructures and Bioimaging (IBB), CNR National Research Council of Italy, 80145 Naples, Italy

**Keywords:** West Nile virus, WNV, mosquito-borne, *Culex*, vaccination strategies, antiviral therapies, Italian vaccines, Iran vaccines, Mediterranean, Middle East, public health surveillance, climate change impact, One Health

## Abstract

**Background**: West Nile Virus (WNV) is a mosquito-borne flavivirus responsible for seasonal outbreaks in temperate and tropical regions, including Europe, the Mediterranean, and the Middle East. Its transmission via mosquitoes, particularly *Culex* species, poses persistent challenges to public health. Despite ongoing efforts, comprehensive prevention and treatment strategies remain limited. **Methods**: A comprehensive search of peer-reviewed literature, clinical trials, and government surveillance data from Italy and Iran was conducted using PubMed, Scopus, Web of Science, and supplementary web-based resources. Inclusion criteria focused on molecular studies of WNV, vaccine and antiviral drug development, and regional outbreak reports. **Results**: WNV transmission is influenced by climatic conditions, as well as vector distribution and ecological patterns. While human vaccines are currently under development, only veterinary vaccines yielded promising but still limited evidence of effectiveness. Notably, therapeutic measures are currently limited to supportive care, whereas investigational antiviral drugs are in early-stage trials. Interestingly, Italy demonstrates robust surveillance with regular reporting of outbreaks, whereas data from Iran indicate that despite a widespread serological footprint, especially in southern and southwestern provinces, the reported clinical impact on humans and animals appears comparatively less severe. **Conclusions**: Bridging gaps in vaccine availability, therapeutic innovation, and disease monitoring is essential for effective WNV management to prepare for potential severe future outbreaks in Europe and the Middle East. On the other hand, regional differences between Italy and Iran reveal the need not only for tailored public health interventions and enhanced surveillance, but also for sustained investment in research. In our view, collaborative frameworks across Mediterranean and Middle Eastern countries in a “One Health” approach may improve preparedness and response to future WNV outbreaks.

## 1. Introduction

Flaviviruses are arthropod-borne viruses within the *Flaviviridae* family, transmitted primarily by mosquitoes. Among them, West Nile virus (WNV), Dengue virus (DENV), and Zika virus (ZIKV) represent major human pathogens [1]. Collectively, flaviviruses have been responsible for extensive outbreaks worldwide, contributing to an estimated 400 million infections annually and posing a significant global health burden [1].

From a biomedical standpoint, WNV is a neurotropic virus that causes infection by replicating within skin-resident dendritic cells, and spreading to lymphoid organs and, in severe cases, penetrating the blood–brain barrier to infect the central nervous system [2]. The clinical spectrum of the disease is extensive, and although the majority of infections are subclinical, around 20% of patients experience a self-limiting febrile illness, and less than 1% advance to neuroinvasive conditions such as encephalitis, meningitis, or acute flaccid paralysis, which are associated with considerable morbidity and mortality rates.

Diagnostic approaches for WNV and related flaviviruses rely on nucleic acid testing (NAT) and serological assays [1]. However, these methods face notable limitations. NAT sensitivity is constrained by the short duration of viremia and low viral loads, while serological assays are complicated by antibody cross-reactivity among DENV, WNV, and ZIKV [3], often leading to misdiagnosis and inaccurate differential results. These diagnostic challenges underscore the need for improved technologies and strategies to enhance accuracy and reliability [3]. At present, human treatment alternatives are confined to supportive care, as no specific antiviral agents have effectively progressed from preclinical trials to clinical licensure. Mosquito control strategies generally impose a low financial burden; however, their impact is transient, as mosquito populations recover quickly and require repeated interventions to contain outbreaks [4].

Because there is not a specific therapy, healthcare organizations experience substantial operational and systemic pressures during periodic epidemics. Since there is not a licensed human vaccine yet, prophylaxis still depends on integrated vector management, public health education, and screening blood and organ donors [5]. However, several next-generation vaccine candidates, such as those using virus-like particles (VLPs) and mRNA platforms, are now being tested in clinical trials. Nevertheless, it is still very important to improve global readiness against this new zoonotic threat by strengthening international cooperation and data exchange [6].

Since its introduction into the United States in 1999, WNV (recently reclassified as *Orthoflavivirus nilense* [7]) has become endemic [8]. Surveillance has traditionally relied on mosquito pool testing, a method that is both resource-intensive and time-consuming. In this context, wastewater-based epidemiology (WBE) has emerged as a promising complementary approach, already validated for other orthoflaviviruses such as DENV [9]. This methodology operates on the premise that pathogens or their metabolic biomarkers excreted by infected individuals, through feces, urine, or other bodily fluids, enter the sewage system, creating an aggregated pool that reflects community-level infection dynamics [10]. Interestingly, recent studies have demonstrated that WNV RNA can be detected in wastewater samples during periods of active viral circulation within communities [8]. Using highly specific and sensitive droplet digital reverse-transcription polymerase chain reaction (ddRT-PCR) assays, detections in wastewater solids have been shown to correspond with seasonal case occurrence and geographic distribution. In one study [8], WNV was identified in nine wastewater samples across three U.S. locations with known clinical cases, with positivity rates ranging from 3.3% to 13%. These findings suggest that WBE could serve as an effective sentinel surveillance tool, particularly in regions lacking extensive mosquito or clinical testing infrastructure [8].

Since the initial identification in Uganda in 1937 [11], the virus has evolved into a globally distributed disease and is responsible for seasonal outbreaks in different temperate and tropical regions, including Europe, the Mediterranean, and the Middle East, where WNV infection remains a pressing public health concern, with outbreaks reported in both rural and urban settings [12,13]. The Mediterranean and Middle East regions present unique challenges, shaped by climatic conditions [14], migratory bird patterns [15], and mosquito habitats [16]. Italy and Iran represent two pivotal case studies for analyzing the eco-epidemiology of WNV in the Eurasian region. Both countries lie along major migratory flyways [17,18,19] and host ecological niches that facilitate viral persistence and reintroduction via avian movement. However, their epidemiological trajectories and public health responses reveal distinct challenges and adaptive strategies.

In Italy, WNV has become endemic, with recurring seasonal outbreaks primarily in northern regions such as Emilia-Romagna and Veneto [20], patterns shaped by climatic warming and agricultural irrigation systems that promote mosquito breeding, while in Iran [21], widespread serological evidence of exposure, is observed, yet relatively few reported clinical cases suggest silent viral circulation among avian and equine hosts under semi-arid conditions. This comparative assessment offers valuable insight into how climatic variability, ecological features, and surveillance capacity shape WNV epidemiology, control, and prevention.

The following sections of this review examine the epidemiological profiles, national responses, and historical outbreak patterns in both countries, while emphasizing the importance of integrated “One Health” approaches [22] to mitigate transmission and anticipate future challenges under evolving environmental pressures. We also discuss the status of therapeutic and vaccination efforts, including challenges in WNV vaccine development, potential drug candidates, molecular aspects of WNV biology, and the role of mosquito transport in viral dissemination.

### 1.1. WNV Lineages

WNV is divided into several genetic lineages, but Lineage 1 and Lineage 2 are most relevant to human and animal health. In fact, Lineage 1 has triggered widespread outbreaks across Africa, Europe, the Middle East, Asia, Oceania, and North America [23]. Although Lineage 2 was long regarded as a relatively mild strain limited to sub-Saharan Africa, this perception has shifted in recent decades as the lineage has spread to new regions and exhibited enhanced pathogenicity [24]. Since the mid-2000s, Lineage 2 WNV strains have caused severe neuroinvasive outbreaks in humans and horses, especially in regions such as Hungary, Greece, and Italy. As a result, virulence-linked mutations (e.g., NS3-249 Proline) have been observed in strains belonging to both Lineage 1 and Lineage 2. The two lineages differ genetically by roughly 20–25% at the nucleotide level. Importantly, certain genetic variations between WNV lineages can change the viral protein structure, which affects immune detection. A key example is the envelope glycoprotein, in which the E glycoprotein of virulent Lineage 1 strains, such as NY99, contains a glycosylation site absent in several attenuated strains, and from a vaccine perspective, such differences are double-edged, as on the one hand, they enable the use of non-glycosylated, attenuated strains as live vaccine candidates [25]. On the other hand, they require broad immunogenicity to ensure protection regardless of whether the envelope protein carries a glycosylation site. Moreover, domain III of the E protein contains critical neutralizing epitopes and is less accessible or altered in some lineage 2 viruses compared to lineage 1. Consequently, an immune response raised against one lineage may neutralize the other less efficiently. However, immunity against one lineage can cross-neutralize the other, reflecting WNV’s classification as a single serotype [26].

For example, horses immunized with an inactivated lineage 1 WNV vaccine, derived from a 1999 NY lineage 1 strain, were protected from lethal challenge with a neurovirulent lineage 2 strain. Similarly, a canarypox-vectored recombinant vaccine expressing WNV lineage 1 pre-membrane (prM)/E glycoproteins completely protected horses against a virulent challenge of lineage 2 [27]. Overall, the immunological and economic challenges hindering WNV vaccine development are further compounded by the genetic diversity between Lineage 1 and Lineage 2, as differences in viral protein structure and pathogenicity demand broad and durable immunogenicity to ensure effective protection across strains.

### 1.2. Climate Changes

Mosquito breeding sites in most parts of Iran are limited due to the arid to semi-arid climate. However, there are also places, such as river valleys and irrigated fields, that can become habitats for mosquitoes [28]. For example, Khuzestan province in southwestern Iran presents a hot, dry climate but broad marshes and rivers; consequently, it exhibits one of the highest WNV levels in the country [29]. However, drought conditions may decrease mosquito breeding sites and suppress WNV transmission. Prolonged drought drives birds and mosquitoes to interact more by concentrating them around the remaining water sources, paradoxically increasing local transmission risk [30]. Overall, unpredictable rainfall patterns and shifting seasonal temperatures create unstable transmission dynamics, which allow the virus to circulate one year and virtually disappear the next year in the same region [31]. Similar climate-driven patterns have been observed in Europe, particularly in Italy. The country’s warm summers create seasonal opportunities for WNV transmission each year [32]. In 2018, Italy experienced unusual climate conditions, including warm April temperatures along with periods of heavy rainfall [33]. Due to these changes, WNV spread to birds earlier than expected and caused a severe summer outbreak. In 2022, on the other hand, spring was dry and lasted into early summer. Then, sudden heavy rainfall and flooding events in July facilitated the formation of suitable habitats for mosquito breeding and helped WNV spread more widely across new ecological areas [31]. Overall, the unpredictable impact of climate variability on mosquito breeding and WNV transmission further complicates vaccine development and public health planning, as the unstable dynamics of outbreaks make large-scale efficacy trials, cost-effectiveness assessments, and long-term prevention strategies even more challenging.

## 2. Methods

A comprehensive methodology was implemented to perform a thorough evaluation of the research literature. This included comprehensive data collection from peer-reviewed literature, clinical trial repositories, and government surveillance data. These sources were critically analyzed to identify key themes, knowledge gaps, and emerging trends, and to develop a comprehensive synthesis that provides a complete understanding of molecular studies, vaccine and antiviral development, as well as the regional outbreak patterns of West Nile virus. First, a comprehensive search was conducted using PubMed, Scopus, Web of Science, and Google Scholar, complemented by government surveillance data platforms and supplementary web-based resources to ensure the inclusion of non-peer-reviewed literature and up-to-date outbreak information. The literature search used terms such as “West Nile virus,” “WNV,” “molecular epidemiology,” “vaccine development,” “antiviral therapy,” “clinical manifestations,” “outbreak reports,” and included location-specific terms “Italy,” “Iran,” “Mediterranean,” and “Middle East”. Specific inclusion and exclusion criteria were applied during the study selection process. Inclusion criteria encompassed peer-reviewed studies published in scientific journals that investigated the molecular characteristics of WNV, including analyses of its genomic, proteomic, or structural features, as well as reports from clinical trials or preclinical laboratory studies evaluating candidate vaccines or antiviral agents directed against the virus. Exclusion criteria involved the omission of non-scientific sources such as newspaper articles, opinion pieces, or reports lacking empirical data, together with studies not specifically addressing WNV, for instance, research on other flaviviruses unless a direct and explicit connection to WNV was established. Withdrawn or retracted studies were excluded. The search encompassed literature published between 1970 and 2025 to capture both foundational and contemporary advancements. Second, an initial screening process was performed to eliminate irrelevant documents. Only empirical studies, observational studies, clinical trials, molecular investigations, outbreak reports, conceptual reviews, and government surveillance data were included, ensuring a balanced representation of different evidence types relevant to WNV. A total of 231 references were ultimately selected for inclusion in the final research library. Among the selected references, about 40% were published within the last five years (2020–2025), reflecting the growing recent research interest in WNV epidemiology, molecular investigation, and countermeasure development.

## 3. Molecular Biology of West Nile Virus

### 3.1. Viral Structure and Genome

**Figure 1:**
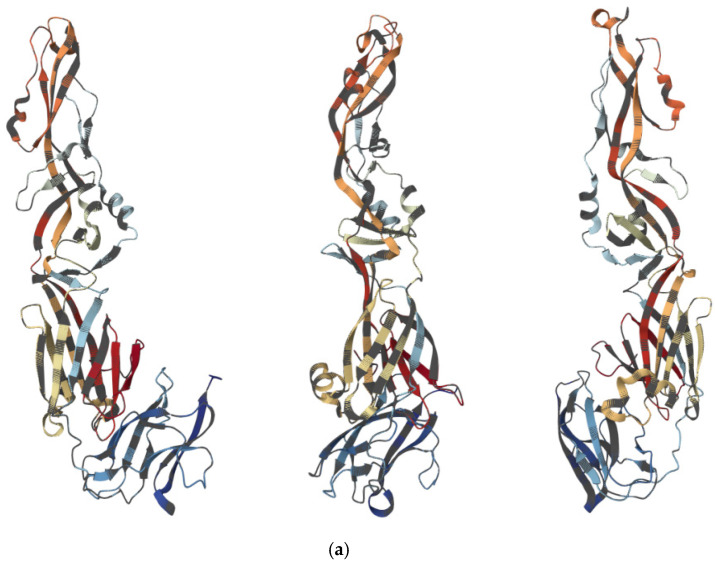

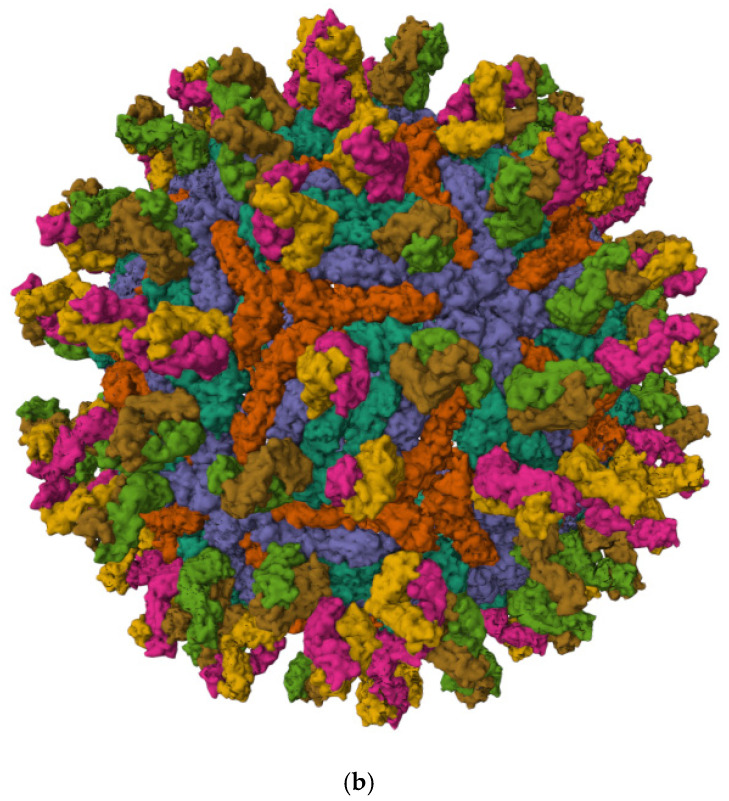
(**a**) Three-dimensional views of the structure of the WNV envelope glycoprotein. (The structure corresponds to PDB ID: 7E4K, freely available online at https://www.rcsb.org/3d-view/7E4K/1, accessed on 24 November 2025). The crystallographic structure of the E glycoprotein, shown in this figure, has a total molecular weight of 43.63 kDa, comprising 3043 atoms and includes 403 modeled residues, organized into a single unique protein chain. (**b**) Three-dimensional view of the WNV virion showing the spatial organization of the envelope protein on the viral surface. The E proteins, embedded in the lipid envelope, are highlighted in purple. (The structure corresponds to PDB ID: 3IYW, freely available online at https://www.rcsb.org/3d-view/3IYW/1, accessed on 19 December 2025).

WNV is a positive-sense, single-stranded RNA virus in the *Flavivirus* genus [34] with a genome of approximately 11 kilobases in length, consisting of a single open reading frame (ORF) flanked by untranslated regions (UTRs) at the 5′ and 3′ ends [35]. The single ORF encodes a polyprotein that is cleaved by host and viral proteases into ten distinct viral proteins [36]. Host proteases contribute to specific cleavage events, while the viral NS2B-NS3 serine protease complex carries out the majority of processing at non-structural protein junctions, ensuring proper maturation of the viral polyprotein [37]. WNV proteins include three structural (capsid (C), prM, and envelope (E)) and seven non-structural (NS1, NS2A, NS2B, NS3, NS4A, NS4B, and NS5) proteins [38]. The envelope (E) protein is the main protein of the virion surface (Figure 1).

E glycoprotein mediates viral attachment and entry by binding to cellular receptors. It is classified as a class II viral fusion protein, a category of proteins that mediate membrane fusion triggered by low pH. After the virus is endocytosed, the acidic pH in endosomes triggers conformational changes in the E protein that drive fusion of the viral envelope with the endosomal membrane, leading to nucleocapsid release into the cytoplasm [39,40]. As the virion transits through the trans-Golgi network (TGN), prM is cleaved by a host furin protease into the mature M protein. This cleavage triggers a major structural rearrangement of the E proteins, leading to the formation of the mature, infectious virion [41,42]. The capsid (C) protein binds to the viral RNA genome to form the nucleocapsid core, which packages the RNA into an icosahedral capsid inside the lipid envelope [43].

Inside the cell, NS1 exists as a dimer and is thought to assist in the assembly of the viral replication complex on endoplasmic reticulum (ER) membranes. While extracellularly, NS1 is secreted as a hexamer and is highly immunogenic by interfering with complement activation and toll-like receptor signaling [44]. The NS2A protein is a compact, multifunctional protein that plays a role in replication, virion assembly, and the modulation of the innate immune response, especially by suppressing interferon-β responses, which can prevent apoptosis of infected cells [45]. NS2B serves primarily as an essential cofactor for the NS3 protease. NS3 is a large, multifunctional enzyme, with its N-terminal region of NS3, together with cofactor NS2B, forming a serine protease that cleaves the viral polyprotein. On the other hand, the C-terminal region of NS3 has helicase/NTPase activity, which unwinds RNA secondary structures and powers replication of the viral genome [46,47]. NS4A and NS4B are small membrane proteins that contribute to the membrane rearrangements of the ER necessary for viral replication. They help form vesicle packets through invaginations of the ER membrane, where RNA replication occurs, and are also involved in suppressing the host’s interferon response and other antiviral signaling [48].

**Figure 2:**
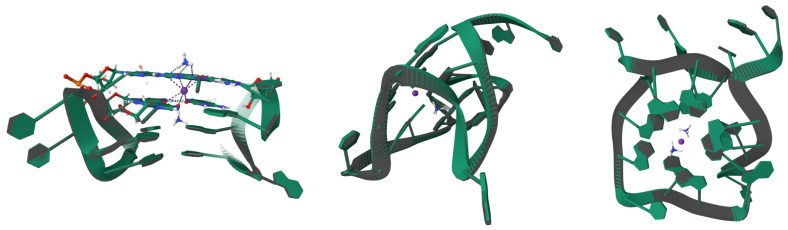
Three-dimensional views of the NS5-B RNA G-quadruplex structure from the West Nile virus genome. (PDB ID: 8UTG. Structure freely available at: https://www.rcsb.org/3d-view/8UTG/1, accessed on 24 November 2025). A K^+^ ion (purple) is involved in G-quadruplex formation within the quartets, and in the crystallographic structure, additional ammonium ions also contribute to the stability of the complex.

Finally, NS5 is the largest and most conserved WNV protein with two enzymatic domains essential for RNA replication. The N-terminal domain of NS5 has a methyltransferase activity responsible for adding a 5′-methylated cap to the viral RNA, which is needed for mRNA stability and to mimic host mRNAs. The C-terminal domain of NS5 is the RNA-dependent RNA polymerase (RdRp) that synthesizes new genomic RNA copies [49,50]. In addition, recent studies have identified potential G-quadruplex (G4 [51,52]) structures within the WNV NS5 region (Figure 2). A high-resolution structure of the NS5-B quadruplex revealed two stacked tetrads stabilized by a stacked triad and transient noncanonical base pairing [53]. This expands the landscape of solved RNA quadruplexes and highlights their potential role as conserved antiviral targets within *Orthoflavivirus* genomes.

### 3.2. Life Cycle Within Hosts

**Scheme 1:**
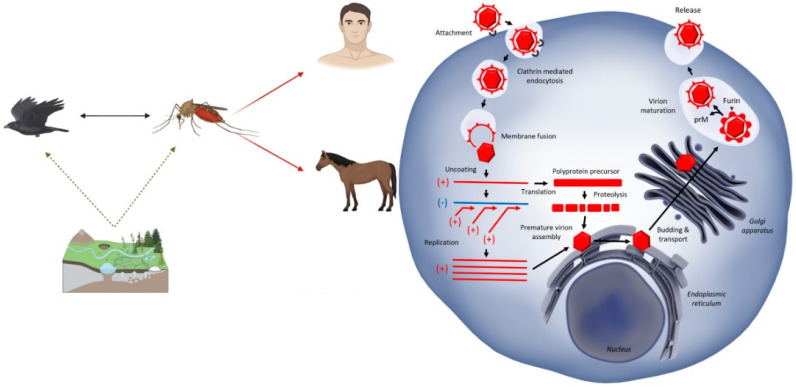
Schematic representation of the West Nile Virus life cycle. The virus is maintained in a mosquito–bird transmission cycle, with occasional spillover to humans and horses, which act as dead-end hosts. Image adapted from Figure 1 of Ref. [57], and Figure 1 of Ref. [58].

WNV initiates infection by attaching to the host cell surface via its envelope E glycoprotein (Figure 1), which recognizes specific cell receptors. Studies have shown that WNV can bind to integrins (like α_v_β_3_), lectins (DC-SIGN and DC-SIGN-R), and phosphatidylserine-binding receptors [54,55]. Once attached, WNV particles enter the cell through receptor-mediated endocytosis, primarily via clathrin-coated vesicles, as the cell membrane invaginates to form an endosome containing the virion (Scheme 1) [56].

As the endosomes mature and pH drops, the WNV E glycoprotein undergoes dramatic conformational changes. E proteins that initially exist as dimers on the virion surface rearrange into extended trimeric structures and lead to the fusion of the viral envelope with the endosomal membrane. As a result, the viral nucleocapsid is released into the cytoplasm. The capsid protein then disassembles and uncoats the viral RNA genome, making it available for translation [59]. Following release into the cytoplasm, the +ssRNA genome of WNV functions directly as mRNA for translation of the viral polyprotein. As previously mentioned, cleavage of the polyprotein into functional products is carried out by both viral and host proteases. These cleavages occur co- and post-translationally: as soon as the polyprotein’s C-terminus emerges into the lumen, host signalase mediates cleavage, and once NS3, together with its cofactor NS2B, are translated, they initiate cleavage at additional junctions [36,60]. When translation and polyprotein processing are complete, structural proteins become embedded in the endoplasmic reticulum, while replication proteins localize to the cytosol to amplify the genome and generate new virus particles [61].

### 3.3. Mechanisms of Replication and Immune Evasion

WNV (like other flaviviruses) induces the formation of ER membrane invaginations or vesicle packets. Within these protected microenvironments, viral RNA replication proceeds efficiently and remains shielded from many host defenses [62]. The non-structural proteins, once cleaved, assemble to form the viral replication complex. Interestingly, the NS5 protein, the core RdRp, copies the genome by initiating the synthesis of a complementary negative-strand RNA using the genomic positive RNA as a template. This minus-strand is quickly complemented by NS5, synthesizing new positive strands from it. Overall, replication generates a pool of genomic positive-strand RNA that serves both as the genetic material for progeny virions and as mRNA for further translation [61]. Assembly occurs on the cytoplasmic side of the ER. The C protein binds to a viral RNA genome to form the nucleocapsid core. These nucleocapsids then associate with regions of the ER membrane where the structural proteins prM and E have accumulated. The virion assembles by budding into the lumen of the ER, acquiring its lipid envelope along with the embedded structural proteins in the process. In the arrangement, the fusion loops of the E proteins are covered by the prM protein, which is critical to prevent the virus from low-pH-triggered conformational changes and fusing with cellular membranes prematurely while it is still inside the host cell’s secretory pathway [63]. These newly formed, ER-luminal particles are immature and non-infectious. They are transported through the cell’s secretory pathway from the ER to the TGN, where the mildly acidic environment triggers a structural rearrangement of the prM-E heterodimers on the virion surface. This rearrangement exposes a cleavage site on the prM protein, which is then recognized and cut by a host cell protease called furin. In particular, furin turns prM into the mature M protein [64]. This process renders the virion infectious by exposing the E protein in the correct conformation for next-cell attachment. Ultimately, the mature virions are carried in secretory vesicles to the plasma membrane and then fuse with the plasma membrane, releasing the viral particles outside the cell via exocytosis [65,66].

## 4. From Transmission to Disease

### 4.1. Role of Mosquito Vectors (With a Focus on Culex Species)

Mosquitoes of the genus *Culex* are the principal vectors of WNV, especially the *Culex pipiens* species [67]. While WNV has been detected in dozens of mosquito species, only a few *Culex* species drive most outbreaks. Environmental factors can greatly influence *Culex* mosquito abundance and WNV amplification and lead to various distributions of the species. *Culex* mosquitoes breed in stagnant water (often with high organic content), such as rain barrels, storm drains, rice fields, or marshes. Warm temperatures and standing water are ideal for their reproduction, so WNV vector populations and virus transmission typically peak in late summer in temperate regions [68]. *Culex pipiens* (the common house mosquito) thrives in temperate urban and suburban settings, *Culex quinquefasciatus* favors warm climates and urban areas, and *Culex tarsalis* is typically found in rural wetlands and irrigated agricultural regions [69,70,71]. While *Culex pipiens* is the principal vector globally, in southern Spain and parts of the western Mediterranean, *Culex perexiguus* plays a decisive role in amplifying the virus among bird populations [72]. Meanwhile, *Culex modestus* is often associated with wetlands and marshes in countries like France and Greece [73]. In Italy, the most common vectors are mosquitoes belonging to the *Culex pipiens* complex, followed by *Ochlerotatus caspius* and *Culex modestus* as secondary vectors [74]. In Iran, *Culex pipiens* was similarly the most common vector of WNV, followed by *Culex sitiens* and *Culex theileri* [75].

### 4.2. Zoonotic Transmission Cycles Involving Birds

*Culex* mosquitoes are typically ornithophilic and prefer to feed on birds. Birds are the natural reservoir hosts of the virus, and when a *Culex* mosquito feeds on a bird that has a high level of WNV in its bloodstream, the mosquito can become infected [76]. The virus replicates in the mosquito and eventually invades the salivary glands. Approximately a week or more after that infectious blood meal, the mosquito can transmit WNV to another host during a subsequent bite [77]. Many *Culex* species feed primarily at dusk and during the night, which is when most transmission to birds (and humans) occurs. Humans and other mammals (such as horses) are incidental “dead-end” hosts for WNV. Although people and horses can be infected through the bite of an infected mosquito and may develop illness, they typically do not produce a viremia sufficient to infect new mosquitoes and therefore do not contribute to sustaining the transmission cycle [38].

### 4.3. Human Infection: Symptomatic vs. Asymptomatic Cases

Approximately 80% of WNV infections are asymptomatic, leading to substantial underreporting, since such cases are typically identified only through blood donor screening or serosurveys rather than clinical illness [78]. On the other hand, around 20% of infected individuals develop a symptomatic illness, typically a mild to moderate febrile syndrome known as West Nile fever. Patients usually experience an acute febrile illness with symptoms such as fever, headache, fatigue, and musculoskeletal pains. Other signs can include a transient rash (often maculopapular) and gastrointestinal symptoms like nausea, vomiting, or diarrhea. Some cases also less frequently report swollen lymph nodes or eye pain. West Nile fever is generally self-limited and usually resolves within days to a few weeks. However, even in mild disease, lingering fatigue or weakness is not uncommon and may persist for several weeks following the acute illness [79].

### 4.4. Neurological Complications and Mortality Rates

Fewer than 1% of all infections (approximately 1 in 150) progress to severe neuroinvasive disease. This serious form may manifest as meningitis, encephalitis, or acute flaccid paralysis. Older adults and individuals with weakened immune systems face a significantly higher risk of developing neuroinvasive complications [80]. Notably, nearly all WNV fatalities occur in neuroinvasive cases (in 2023, 98% of WNV deaths were in patients with neuroinvasive disease) [81]. In this frame, West Nile encephalitis, an infection of the brain parenchyma caused by WNV, is the most common neuroinvasive presentation, accounting for roughly 60% of West Nile Neuroinvasive Disease (WNND) cases. Clinically, encephalitis is marked by encephalopathy (manifesting as confusion or decreased consciousness), often with focal neurological deficits, seizures, or tremors. West Nile encephalitis is considered the most severe form of WNVD because it often causes prolonged hospitalization [82]. An estimated 10–15% of encephalitic cases are fatal, with higher mortality in the elderly [83].

West Nile meningitis represents about 20–30% of WNND cases. Clinically, it presents with symptoms such as headache, stiff neck, photophobia, and fever, often without the profound confusion seen in encephalitis. While meningitis requires hospitalization, patients with isolated West Nile meningitis generally have a better prognosis than those with encephalitis, and the case fatality ratio for pure West Nile meningitis is typically well under 5% [84,85]. WNV can invade the anterior horn cells of the spinal cord, leading to acute flaccid paralysis (AFP), often termed a poliomyelitis-like syndrome. This manifestation is present in about 5–10% of neuroinvasive cases [86,87]. It is characterized by the sudden onset of asymmetric weakness or paralysis of one or more limbs, often without loss of sensation. It may occur in isolation or alongside encephalitis or meningitis, with about 24% of WNND patients with AFP also showing encephalitic or meningeal symptoms. Respiratory muscle involvement (e.g., diaphragmatic paralysis due to phrenic nerve involvement) has a high risk of complications and death since it can lead to respiratory failure requiring ventilatory support. Furthermore, many patients with WN polio-like syndrome are left with residual paralysis or muscle weakness, as motor neuron damage may be permanent [88,89].

## 5. The Epidemiological Landscape: The Mediterranean and Middle East Overview

### 5.1. Case Study: Italy vs. Iran

The comparative analysis of the prevalence of WNV in two pivotal epidemiological hubs, Italy and Iran, illustrates the pattern of this disease in the Mediterranean and the Middle East. Italy, as a southern country in Europe and a key hotspot, has experienced a number of outbreaks, particularly seasonal ones [32]. Similarly, Iran shows an elevated rate of seroprevalence in humans and animals, especially in the southern parts of the country [90]. The selection of these two countries is also crucial due to their critical position in the African-Eurasian migratory bird flyways. Additionally, these countries have high potential for sustaining the virus transmission cycle, due to the presence of *Culex* mosquito vectors and suitable climatic conditions. Epidemiological data presented in this section refer to confirmed cases of WNND. These data are used as reliable indicators of WNV activity, since most WNV infections remain asymptomatic. In particular, among European nations, Italy has a high incidence of WNV. In fact, as of 24 October 2025, Italy has reported 767 human cases of WNV infection, including 366 WNND cases and 69 deaths. The majority of cases were recorded in Lazio, Campania, Lombardy, Piedmont, and Veneto, with viral circulation detected across 78 provinces in 18 regions [91]. The number of WNND cases reported in 2024 was 272, and in 2023 was 190 [92]. These cases are commonly reported in the summer and at the beginning of fall. *Culex* mosquitoes, as the primary vectors, have a boosted life cycle due to high temperatures, and their number increases significantly during this period. In addition, heat can decrease the extrinsic incubation period and elevate the rate of incidence of the WNV. Also, the migration of birds, the main reservoirs of the disease, increases and facilitates the outbreak of the WNV [93].

Between 2008 and 2011, Italy reported seven deaths due to WNV infection [94]. Also, from 2012 to 2020, there were 60 deaths attributed to WNV [94]. Significant surges in mortality were observed during two major outbreaks, with 49 deaths recorded in 2018 and 48 in 2022 [95,96]. In more recent years, Italy recorded 4 deaths in 2019, 5 in 2020, none in 2021, 29 deaths in 2023, and 21 deaths in 2024 [96]. Overall, fatalities were mostly in northern Italy, particularly in Emilia-Romagna, Veneto, and Lombardy, with fewer deaths occurring in the country’s central and southern regions [32].

In Iran, in recent years, although documented human cases of WNND have remained rare, findings from serological assessments suggest that significant levels of exposure are continuing within the population [97]. This transmission cycle is sustained by *Culex* mosquito populations, whose numbers and viral transmission efficiency increase under high summer temperatures [98]. Additionally, WNV is frequently reintroduced into local bird-mosquito cycles, particularly in areas with a high concentration of wetlands [28]. Previous studies have demonstrated geographical variation in human WNV exposure across Iran. Southern and southwestern provinces, such as Fars, Khuzestan, and Hormozgan, exhibited the highest seroprevalence rates, more than 20% in several studies. The warm and humid conditions in the south provide suitable habitats for *Culex* mosquitoes to have longer breeding seasons and higher transmission rates. Moreover, wetlands and migratory bird routes in regions like Khuzestan and Bushehr facilitate virus circulation between birds, mosquitoes, and humans [90,99]. In contrast, northern provinces, including Golestan, Gilan, and Qom, reported low seroprevalence [21]. These provinces have cooler climates that limit mosquito activity and reduce the period of virus transmission. From 2019 to 2024, Iran recorded very low numbers of confirmed WNV cases each year, with no widespread WNV epidemic occurring in the country. Although serological surveillance indicates WNV circulation in many Iranian provinces, most identified human infections were asymptomatic and detected by antibody tests rather than clinical case notifications [97].

### 5.2. National Health Strategies

#### 5.2.1. Strategies Adopted in Italy

The Italian government has changed its strategies to control WNV over the years. After finding the new cases in the late 1990s, the movement of horses was banned within a 700 km^2^ area until the activity of vectors declined to minimal levels. Also, the targeted area was segmented into multiple zones to improve control measures [100]. In general, the main focus was on monitoring animals, particularly horses and birds, given their high susceptibility to WNV, with the virus often causing severe and sometimes fatal neurological disease in horses, thereby making them one of the most affected non-human species. This approach was a reactive strategy, as the disease had not yet spread extensively. In 2008, following the WNV epidemic in northern Italy, the government implemented an integrated surveillance strategy aimed at controlling the disease in humans, animals, birds, and mosquitoes. Furthermore, a vaccination program for horses was initiated in 2009 and subsequently expanded to other regions of the country [101]. Veterinary surveillance was a strategy for early detection of cases in animals, especially sentinel horses and birds. This strategy was composed of two parts: active surveillance (systematic searching) and passive surveillance (spontaneous reporting) [102]. In entomological surveillance, the use of CO_2_-baited and BG-Sentinel traps helped collect a considerable number of *Culex pipiens* mosquitoes, which were then tested using Reverse Transcription Polymerase Chain Reaction (RT-PCR). This system helped provide an early warning signal to detect viruses in mosquitoes before they appear in animals and humans [103].

Human surveillance involved reporting acute meningoencephalitis or other neurological disorders that might be due to WNV. In this method, clinical specimens, including cerebrospinal fluid and blood, were analyzed by using serological markers, such as IgM and IgG. After the emergence of WNV in Italy, it became crucial to prevent the transmission of this virus through blood transfusions. In this frame, nucleic acid testing is an effective method for detecting viral genetic material in the blood, which helps prevent the spread of the virus through blood donation [104]. The combination of the mentioned approaches played an important role in controlling the disease [105].

More recently, monitoring efforts for the disease intensified, and regions were classified according to reports of new cases, distinguishing between affected areas and those under surveillance. Surveillance regions referred to areas adjacent to affected areas, which were considered to be at high risk of future outbreaks. Public awareness also increased, which helped to reduce the likelihood of disease outbreaks [94]. A “One Health” approach has proved effective through exchanging information about the spread of WNV among humans, animals, and mosquitoes. This collaboration was between veterinary services, public health organizations, and environmental institutions, which could enhance the impact of preventive strategies [106]. Additionally, another warning system, utilizing data from Earth Observation, specifically Land Surface Temperature, Normalized Difference Vegetation Index, and Surface Soil Moisture, was able to predict the outbreak of WNV two weeks earlier with an accuracy of 84% [107].

#### 5.2.2. Strategies Adopted in Iran

Historically, WNV did not figure prominently in Iran’s public health priorities; however, this has been changing in light of accumulating evidence of its circulation in the country. Iran’s Ministry of Health and research institutions have taken steps to better understand and control WNV in recent years. Firstly, they established a surveillance and diagnostics system. In the 2010s, Iran’s national reference labs began incorporating WNV into testing panels for encephalitis of unknown origin [108]. Additionally, Iran’s primary defense relies on vector control and public education, emphasizing the importance of raising awareness about WNV and its transmission through mosquito bites [109].

### 5.3. Historical Outbreaks and Containment

#### 5.3.1. The WNV Situation in Italy

**Figure 3:**
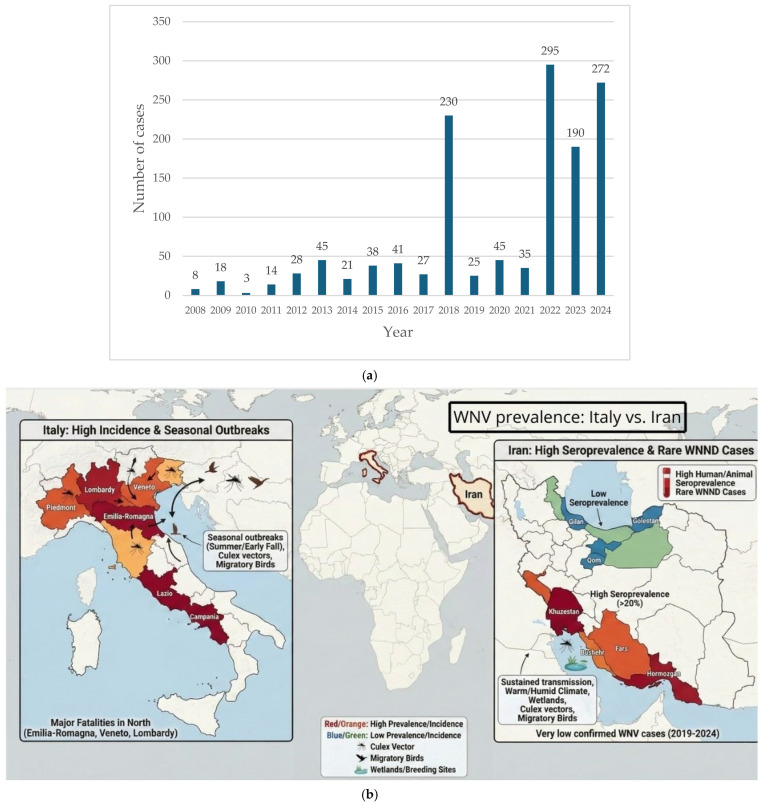
(**a**) Annual number of reported West Nile Neuroinvasive Disease cases in Italy from 2008 to 2024. Each bar represents the total number of reported WNND cases for a given year, with the *y*-axis indicating the number of cases and the *x*-axis representing the years. The data highlights the trend and fluctuations in reported cases over the period, providing insights into the temporal dynamics of WNND in Italy [92,110,111,114,115,116,118,120,122,123,124]. (**b**) Map illustrating comparative prevalence of WNV in Italy and Iran, highlighting high-prevalence regions and key epidemiological features. The map was created by the authors based on epidemiological data reported in [21,32,90,92,94,95,96,97,99] and edited in Canva online program (https://www.canva.com/, accessed on 24 November 2025).

In the summer of 1998, Italy experienced its first officially recognized WNV outbreak among horses in the Tuscany region. During this period, 14 cases of the disease were detected in horses, with a case-fatality rate of 43% [100]. Approximately a decade later, in 2008, eight people in northern Italy were diagnosed with neuroinvasive WNV, and in 2009, 18 more people were diagnosed with the same disease [110,111]. Moreover, 794 horses were affected in northern Italy, with about 32 showing signs of neurological symptoms. In addition to humans and horses, WNV was confirmed in other animal populations, including magpies (*Pica pica*), carrion crows (*Corvus corone*), and rock pigeons (*Columba livia*) [112]. By studying the spread of WNV in mosquitoes, mosquito surveillance in Emilia-Romagna detected active transmission, particularly among *Culex pipiens* [113]. Although there were only three cases of WNND in 2010, mostly in the north, the disease expanded throughout 2011 in Sardinia, one of the two largest islands in Italy [114]. In the summer of 2011, Sardinia experienced an outbreak of the disease with six hospitalized neuroinvasive cases and four deaths [115]. After this expansion, Italy reached a peak of 28 WNND cases with two fatalities in 2012, and 14 RNA-positive blood donors, which indicated silent circulation of the virus [116]. Between 2013 and 2017, the infection continued to circulate at manageable levels in Italy, with limited infections observed in mosquitoes, birds, and humans. In contrast to previous years, Italy experienced a sharp rise in WNV activity in 2018 [116]. Compared to previous years, warmer spring conditions in 2018 contributed to an earlier and more intense spread of the virus among mosquitoes and birds [117]. Consequently, according to Epicenter reports, the number of reported human cases increased from about 24–25 per year during 2013–2017 to 230 cases in 2018 (Figure 3a) [118]. The number of WNND cases in Italy changed annually between 2019 and 2022. The COVID-19 pandemic made it more challenging to monitor and identify cases, as resources and attention were focused on addressing the pandemic [119]. Italy experienced an increase in WNND in 2022, with more than 290 cases. In 2023, fewer than 200 cases were recorded, while in 2024 the number exceeded 270 (Figure 3a). This outbreak was among the largest ever recorded in the country and in Europe, and it was associated with the spread of a new WNV lineage 1 strain [120]. The virus also started spreading in mosquitoes and birds earlier than usual that season (Figure 3b), which gave it more time to spread before control measures were fully effective [121]. This unexpected outbreak demonstrated the urgent necessity of strengthening national surveillance systems and vector control programs.

#### 5.3.2. The WNV Situation in Iran

WNV was first detected in Iran in the 20th century through serological evidence, before any clinical outbreak was recognized. A 1968–1969 survey in northeastern (Khorasan) and southwestern (Khuzestan) Iran found WNV antibodies in 28.4% of samples from the general population [125]. The first confirmed human WNV infections were reported in 1976 in central and southwestern Iran, which were based on antibody surveys rather than observed illness outbreaks [126]. For many years, Iran lacked a centralized surveillance system for WNV and its neuroinvasive form, resulting in minimal and inconsistent annual case documentation. Available data came mostly from research studies and isolated reports rather than official year-by-year records. From 1977 through the early 2000s, Iran reported no recognized WNV outbreaks, because infections were either asymptomatic or misdiagnosed, and surveillance was minimal [127]. The following years provided more insight into the silent but persistent presence of the virus. In 2008–2009, three people from Isfahan got encephalitis and tested positive for WNV in their cerebrospinal fluid through RT-PCR. This was the first confirmed case of WNND in Iran. One of the patients from the 2009 Isfahan cluster ultimately died from the infection, highlighting that serious clinical outcomes may arise even during initial, small-scale outbreaks [108]. In 2010, a study of blood donors in Tehran showed that 5% carried WNV antibodies, despite no symptomatic cases being reported in the capital [128]. In 2011, a nationwide equine survey across 27 provinces found that almost a quarter of horses had neutralizing antibodies, and some IgM-positive cases indicated very recent infections [129]. In 2011–2012, southwestern provinces such as Khuzestan reported even higher exposure, with nearly 70% of horses testing positive [130].

From 2015 to 2016, studies added further confirmation. Around 32,000 mosquitoes collected from northern wetlands were screened, and WNV RNA was found in *Culex pipiens* in Gilan province. While no human cases were identified, the virus’s presence in vectors proved that active cycles were ongoing [75]. The following year, in 2016–2017, a detailed study in Hormozgan province (southern Iran) revealed that 20.6% of the human population had antibodies, and local mosquitoes also tested positive. These findings implied substantial, possibly outbreak-level transmission, even though no official human cases were reported. This gap was related to weak surveillance and underdiagnosis [127]. In 2018, in Sistan and Baluchestan, on the southeastern border, 8.2% of blood donors carried antibodies. In the same year, wild bird and equine studies in northern provinces (Mazandaran, Golestan, Kordestan, and North Khorasan) showed seroprevalence rates of about 14% in birds and 17% in horses. Still, despite widespread circulation, official reports continued to state that no confirmed clinical WNV or WNND cases had been recorded nationally [29] and, as anticipated in Section 5.1, between 2019 and 2024 Iran likewise reported only very low numbers of confirmed WNV cases each year, with no widespread epidemic occurring in the country (Figure 3b).

## 6. Current Status of Vaccination and Therapeutic Efforts

### 6.1. Challenges in WNV Vaccine Development

Antibody-dependent enhancement (ADE) is a phenomenon in which the antibody-virus complexes interact with immune cells, such as monocytes, macrophages, and dendritic cells, promoting Fc receptor-mediated endocytosis. This process leads to increased viral replication and disease severity [131]. In fact, ADE is most likely to occur when pre-existing antibodies, generated in response to a related flavivirus or vaccination, bind to the virus in a sub-neutralizing manner, which increases the severity of a subsequent infection instead of fighting the virus [132]. Cross-reactivity further contributes to ADE among members of the *Flaviviridae* family, which includes Dengue, WN, Zika, Yellow fever, and Japanese encephalitis viruses [133,134]. This family all shares similar envelope glycoproteins, which are the targets for neutralizing antibodies. Due to this resemblance, some antibodies generated against one flavivirus may cross-react with another flavivirus and bind partially in a non-neutralizing manner [135,136].

Another immunological challenge is aging, which is associated with high vulnerability to infections. As people age, certain immune cells, such as B cells, CD4+, and CD8+ T cells, are produced less efficiently, leading to signaling dysregulation and immune dysfunction [137]. Additionally, macrophages exhibit reduced phagocytic activity and impaired cytokine production [138]. This age-related immune deficiency, called immunosenescence, contributes to an increased susceptibility to viral infections among older individuals. Significant economic challenges are hindering the development of a licensed WNV vaccine, despite early trials showing promising results for safety and immune response, as conducting large-scale efficacy trials is prohibitively expensive and complicated due to the unpredictable timing and geographic distribution of WNV outbreaks. Together with the need for multiple doses and the low incidence observed over many years, these factors have raised doubts about its cost-effectiveness, although hospital data from Colorado demonstrate the substantial economic burden of WNV, with initial treatment costs exceeding $20,000 for patients with encephalitis and more than $25,000 for those with AFP [139]. Looking at the bigger picture, this adds up to an estimated $778 million in total hospitalization costs across the U.S. between 1999 and 2012 (as reported at the link https://www.sciencedaily.com/releases/2014/02/140210184713.htm accessed on 24 November 2025). Instead, it may be more cost-effective to target vulnerable populations, such as older adults.

### 6.2. WNV Vaccination Strategies

Against this backdrop of immunological, ecological, and economic challenges, it becomes essential to consider how broader biomedical innovations can contribute to overcoming the obstacles in WNV vaccine development. Vaccines based on nanoparticles and virus-like particles represent a beneficial candidate for safe and effective WNV vaccination. These VLPs naturally assemble into virus-like shapes and display antigens in their native conformation and repetitive patterns, which effectively activate B lymphocytes [140]. By expressing both prM and E proteins, researchers can create virus-like particles that structurally look like real flaviviruses without carrying any genetic material. Notably, WNV VLPs made in insect or mammalian cells have shown protective immunity in animal models [141]. In addition to utilizing WNV structural proteins for VLP formation, researchers are designing mosaic nanoparticles that display critical WNV antigenic domains on a foreign VLP scaffold. One such involves linking the envelope’s domain III (DIII) to bacteriophage AP205 particles. Covering these particles with many copies of DIII increased the exposure to neutralizing epitopes, and immunization with these conjugates triggered WNV-specific neutralizing antibody production in immunized mice [142,143]. Although VLP vaccine production involves technical challenges, the large-scale production of Hepatitis B and HPV VLP vaccines in yeast and insect systems shows their feasibility for industrial-scale production [144]. Bacteriophage VLP conjugates, such as AP205-DIII, could be produced mainly in microbial systems, which are cost-effective [143]. Overall, to meet regulatory standards, a WNV VLP vaccine must consistently produce uniform particles across batches and confirm the absence of residual nucleic acids or infectious particles. One of the regulatory considerations for this approach is its potential to promote a DIVA (Differentiating Infected vs. Vaccinated Animals) strategy. Since VLP vaccines are designed without non-structural antigens, such as NS1, they only generate antibodies against structural proteins. As NS antibodies are generated only after natural infection with wild-type WNV, they are considered a helpful marker to distinguish infected animals from those that were vaccinated, which is beneficial for veterinary monitoring and tracking WNV transmission in immunized populations [145].

RNA-based platforms are produced through cell-free enzymatic transcription and are considered an important candidate for future vaccine development after their utilization against COVID-19 [146]. These formulations provide synthetic mRNA that codes for WNV antigens via lipid nanoparticles and facilitate endogenous synthesis of the viral protein by host cells [147,148]. The internally expressed antigen mimics a natural infection and activates humoral and cellular immune responses. Therefore, mRNA vaccines generate a dual immune response through the translation of intracellular antigens and the presentation of these antigens via MHC class I. This mechanism is necessary for WNV control, as neutralizing antibodies limit viral spread and T-cells destroy infected cells [149]. Additionally, technological progress in mRNA vaccine design has improved the efficacy. For example, influenza and COVID-19 mRNA vaccines produced significant antibody titers even in elderly populations, in which mRNA platforms may reduce immunosenescence associated with WNV vaccination [150]. However, these vaccines still encounter several distribution challenges due to cold-chain demands, which range from −20 °C to −70 °C. Overall, although no mRNA-based vaccine for WNV has reached clinical trials to date, the mRNA platforms against similar flaviviruses, such as Zika and dengue, have shown protective outcomes in animal models, which supports the idea that they could be a valuable approach for WNV vaccine development [146].

DNA vaccines generally utilize a plasmid encoding the WNV prM and E glycoproteins, which are expressed in host cells after intramuscular injection [151]. These engineered plasmids are also non-pathogenic and cannot replicate in animal cells [23]. An early WNV DNA vaccine showed favorable immunogenicity in a Phase I trial through encoding the NY99 strain prM/E under a standard CMV promoter [152]. This strong promoter produces higher expression of the prM and E proteins, which increases the production and secretion of non-pathogenic subviral particles. In a trial of a CMV/R promoter-driven WNV DNA vaccine, three doses produced neutralizing antibody responses in more than 95% of participants, including older adults, with similar antibody magnitude and duration as in younger adults. As a result, DNA vaccination can reduce age-related immunological concerns [153]. Another strength of DNA vaccines is their capacity to include additional antigenic components as needed, since they can be engineered to express an expanded range of WNV proteins, such as NS1 or the capsid, which helps stimulate a broader range of T-cell responses and may promote overall vaccine efficacy [23,154].

The primary limitation of Plasmid DNA vaccines is that they show stronger immunogenicity in mice compared to weaker responses in humans, mainly due to challenges with delivery and transfection. DNA plasmids must enter the cell nucleus, but standard naked DNA injection is inefficient. Therefore, other effective delivery methods are required, such as electroporation to increase cell permeability. Reaching appropriate immune responses often necessitates high doses and invasive delivery methods, which are costly and unsuitable for large-scale application [155,156].

In general, plasmid DNA is affordable to produce in bacterial culture on a large scale and is highly stable; however, no human DNA vaccine has yet been licensed in Europe [157]. Altogether, the convergence of VLP, RNA, and DNA vaccine strategies with broader biomedical innovations demonstrates that, although important progress has been made, substantial challenges in production, regulation, and distribution remain, and the path toward a viable and scalable West Nile Virus vaccine is still uncertain.

#### 6.2.1. Vero-Derived Vaccines in Human Trials

Recent advances in WNV vaccine development have demonstrated promising results in animal models, including horses and mice. However, as noted above, the successful translation of these outcomes into an effective human vaccine has not yet been achieved [158]. Researchers used the NY99-35262 strain to formulate WN-VAX, an inactivated WNV vaccine derived from Vero cells, with the NY99-35262 designation referring to a specific isolate from the 1999 New York outbreak, which was subsequently adapted for laboratory use [159]. After the mice received two doses, the vaccine enhanced neutralizing antibody production, and vaccinated mice exhibited a strong humoral immune response through the production of IgG proteins. Additionally, pre-existing immunity to the Japanese encephalitis virus has been shown to increase the immunogenicity of WN-VAX in mice. Ten weeks after vaccination, seroconversion was fully achieved in mice previously immunized with a vaccine for Japanese encephalitis virus, whereas the seroconversion rate among naïve mice remained at approximately 50%. Overall, these findings support that inactivated WNV vaccines derived from Vero cells are a reliable and safe approach for future vaccine development [159,160].

#### 6.2.2. Weakened WNV Vaccines

Another strategy for WNV vaccine development is to use live-attenuated vaccines based on lineage II WNV strains, which may reduce safety concerns compared with highly virulent lineage I strains, even though lineage II viruses have also caused outbreaks in Europe. WN1415 (referred to as W956) is a molecular clone based on the lineage II B956 strain. In mice, a low dose of 55 plaque-forming units (PFU) stimulated neutralizing antibody responses three weeks after immunization and protected against mortality in approximately 67% of the mice, whereas the vaccinated mice with higher doses were protected entirely [161,162]. Notably, all unvaccinated mice exposed to a lethal dose of the NY99 lineage I WNV strain died, supporting the vaccine’s efficacy.

#### 6.2.3. Chimeric WNV Vaccines

In preclinical studies, a chimeric WNV vaccine using the yellow fever 17D backbone (also known as ChimeriVax-WN) was significantly less neurovirulent than even the standard YF 17D vaccine in both mice and non-human primates [163]. Moreover, an initial chimeric construct (ChimeriVax-WN01) containing wild-type WNV envelope genes showed partial neurovirulence in murine models. However, by introducing three specific attenuating mutations into the WNV envelope at residues E107, E316, and E440, ChimeriVax-WN02, a second-generation vaccine, exhibited reduced neurovirulence [164]. As a result, when vaccine and reference viruses were injected directly into the brain, animals that received ChimeriVax-WN02 had notably lower neuropathologic lesion scores than those given YF 17D. Similarly, a chimeric WNV vaccine constructed on a dengue-4 backbone with a 30-nt deletion in the 3′ UTR (WN/DEN4Δ30) showed lower neuroinvasive potential, and live-attenuated vaccines in general are known to generate long-lasting immunity with a single dose [5]. Both the ChimeriVax-WN02 and WN/DEN4Δ30 vaccine candidates have indicated strong immunogenicity.

In Phase I trials of ChimeriVax-WN02 in adults, 100% of participants aged from 18 to 40 years old developed neutralizing antibodies by 21 to 28 days after a single injection [165]. In one study, most individuals developed high seroconversion at 1 month. One year after 3 doses, most participants still had measurable levels of neutralizing antibodies, and the magnitude and duration of responses in adults aged 51–65 were similar to those in younger adults [153]. Additionally, live WNV vaccines used in animals have shown long-lasting protection. For example, horses generally need two doses of traditional vaccines and an annual immunization. In contrast, a single-dose live chimeric YF/WN vaccine (PreveNile) provides strong protection with just one dose. Overall, these live-attenuated WNV vaccines are genetically stable, and they have shown safety characteristics, such as limited neurovirulence [163,166].

#### 6.2.4. Viral Vector Vaccines

Viral vector vaccines use viruses as carriers, which are generally non-pathogenic. They deliver genetic instructions for WNV proteins, typically the prM/E surface proteins, which trigger the production of protective antibodies [165]. Given these features, the Modified Vaccinia Ankara (MVA) virus is an ideal platform for a WNV vaccine [167]. MVA vector exhibits multiple formats of the E-antigen. This approach provides a safe immune response and produces high levels of neutralizing antibodies, as well as E-specific T cells. It also offers complete protection against lethal challenge from both Lineage 1 and Lineage 2 WNV strains [168]. The MVA-WNV vaccine is a strong candidate against WNV. It is non-replicating, which confirms safety, and its manufacturing is already well established. Additionally, viral vectors efficiently express antigen production within host cells and trigger strong immune responses even at lower doses [167]. All these advantages make them a vital tool when rapid emergency vaccination is required.

#### 6.2.5. Existing Veterinary Vaccines and Implications for Public Health

WNV circulates naturally between birds and mosquitoes, while humans and horses are dead-end hosts [169]. However, vaccination of horses is considered a core equine immunization in endemic regions [170]. Several WNV vaccines have been developed for veterinary use [165]. These include traditional inactivated virus vaccines, recombinant viral-vectored vaccines, chimeric flavivirus vaccines, and a DNA-based vaccine, but no licensed WNV vaccines exist yet for birds or humans. In Europe, three veterinary WNV vaccines are approved: one inactivated (Zoetis’ Equip WNV), one canarypox recombinant (Merial/Boehringer’s Proteq WNV), and one inactivated YF chimera (Merck’s Equilis WNV). These were certified through the European Medicines Agency starting in 2008, as WNV spread into Europe [171]. Inactivated WNV vaccines contain the whole virus that has been killed and formulated with an adjuvant. They induce immunity without the use of live virus and reduce viremia. These vaccines are generally given as an initial two-dose series followed by annual boosters [170]. In addition, recombinant live-vectored vaccines utilize a non-pathogenic virus as a carrier to deliver WNV antigens and are non-adjuvanted with a strong safety profile [165]. The vaccine strain (vCP2017) is a canarypox virus expressing WNV preM/E genes; after injection, it does not replicate in the horse, but it produces WNV antigens to induce immunity [172].

Another vaccine example is the recombinant live-vectored vaccine that utilizes a non-pathogenic viral backbone, such as the canarypox virus [173]. For instance, the Prevenile WNV vaccine is a chimeric WNV vaccine that uses a yellow fever 17D virus backbone expressing WNV structural proteins. This chimeric vaccine was initially licensed in the late 2000s and showed the advantage of a single-dose immunization [23,164]. However, Prevenile was withdrawn from the market in 2010 due to the increased incidence of adverse events in the field. Currently, Equilis West Nile uses a non-replicating chimeric virus. It is a yellow-fever-WNV chimera that is inactivated before formulation. Approved in Europe since 2013, it contains an inactivated YF-WNV chimera adjuvanted with an ISCOM-Matrix to enhance immunity. Like other vaccines, it is given in two doses and annual boosters, and provides at least 12 months of immunity in horses. Likewise, rhesus macaques (*Macaca mulatta*) immunized with a chimeric WNV/Dengue-4 virus (WN/DEN4-Δ30) showed strong immunity with no detectable viremia and full protection on challenge [174,175]. Additionally, formulation of the WN-80E subunit vaccine with the saponin-based adjuvant GPI-0100 generated strong antibody responses in rhesus monkeys. Monkeys vaccinated with either a 1 µg or 25 µg dose developed high neutralizing antibody titers. Approximately half of the vaccinated monkeys also showed WNV-specific T-cell activation, as indicated by lymphoproliferation and cytokine production. In a challenge trial, all vaccinated macaques were protected entirely, and none of the immunized animals developed any viremia after WNV challenge, whereas all control animals had detectable viremia for at least 3 days. This 100% efficacy highlights the potential of subunit vaccines in primates [176].

For horses, Italy currently licenses two main vaccines: the inactivated Equip WNV (Pfizer/Zoetis) and the recombinant Proteq West Nile (Merial) [177]. These vaccines protect against both lineage 1 and 2 WNV strains present in Europe. A 2020 study found that 10 of 60 tested horses (16.7%) in northern Iran (Golestan province) had antibodies against WNV. Wild birds in Iran also show WNV exposure, including migratory species that likely introduce or maintain the virus. However, Iran does not produce its own WNV vaccine for horses and lacks domestic licensure of such products (or at least this is not documented in the international literature) [178]. This situation is probably related to the comparatively lower mortality rates among horses in the country. In general, vaccination produces high levels of neutralizing antibodies and protects horses from developing viremia and disease when exposed to WNV. For example, the Fort Dodge/Pfizer inactivated vaccine was shown to be almost 94% effective in preventing viremia in an experimental challenge study. In that trial, horses received two doses 14 days apart; one year later, 94% of vaccinated horses were protected from WNV viremia, whereas 82% of unvaccinated controls developed viremia [23]. Another study of the canarypox-vectored vaccine demonstrated complete protection against WNV challenge in the vast majority of horses. In a group of 28 horses given two doses of the recombinant vaccine, none of the vaccinated animals developed clinical signs, and all but one horse remained free of viremia after exposure to WNV. By contrast, 80% of the unvaccinated control horses became viremic. Even though these vaccines provide sufficient protection, they show meaningful differences in several fields. For example, the inactivated (Pfizer) vaccine produced a faster initial response, and 100% of horses had detectable IgG by 18 days after the first dose, versus about 38 days for 100% seroconversion with the canarypox vaccine. On the other hand, the canarypox vaccine produced a stronger neutralizing antibody response, showing a longer-lasting effect that remained strong for a full year (65% of horses maintained high neutralizing titers at one year with Merial’s vaccine, compared to 21% with Pfizer’s).

Unlike horses, most domestic mammals are hosts for WNV that rarely develop severe disease. Researchers evaluated the canarypox-vectored recombinant WNV vaccine in dogs and cats. In a controlled trial, animals received two doses 28 days apart. Dogs (*n* = 17) were vaccinated with 10^5.6^ TCID_50_, and cats were assigned to a high-dose group (*n* = 14, 10^7.5^ TCID_50_) or a low-dose group (*n* = 8, 10^5.6^ TCID_50_). Unvaccinated controls included 15 dogs and 11 cats. At 4 months post-vaccine, the dogs and cats were exposed to WNV via bites from infected mosquitoes. Surprisingly, none of the 17 vaccinated dogs developed any viremia, whereas 14 of 15 unvaccinated control dogs became viremic. Among cats, only 1 of 22 vaccinated cats showed a trace viremia post-challenge (and that was in the low-dose group), compared to 9 of 11 control cats that developed viremia [179]. Although veterinarians do not routinely vaccinate pets for WNV, due to extremely rare natural infection in dogs and cats, these results suggest that, if needed, dogs and cats could be protected by vaccination.

Other mammalian models, such as mice and hamsters, are used as preclinical models to determine the effectiveness of the vaccines, as they tend to develop WNV disease. For instance, mice vaccinated with chimeric insect-specific flavivirus vaccines or VLP-based vaccines are challenged with the virus after treatment to evaluate protective outcomes. The most effective vaccines are related to strong antibody production and active T-cell responses, which prevent paralysis and death in these rodents [180]. Additionally, camelids like alpacas and llamas are also susceptible to WNV, and neurologic disease has been reported in some individuals, but no licensed camelid WNV vaccine exists. In a trial, alpacas and llamas received a three-dose schedule of an inactivated WNV vaccine. After the second vaccination, about 90–96% of llamas and alpacas had developed neutralizing antibodies (similar to horses after two doses), and by 3 weeks after the third dose, nearly 100% of alpacas and 97% of llamas were seropositive. The results showed no significant adverse effects in the 84 camelids vaccinated. The data showed that camelids need a third dose to achieve the same level of immune protection observed in horses following the standard two vaccinations. Testing these vaccines on different types of mammals, such as monkeys and horses, provides beneficial information to protect susceptible mammals and enables more effective responses when outbreaks occur [181]. An overview of current WNV vaccine candidates, covering platforms, mechanisms, and development status, is provided in Table 1.

### 6.3. Potential Drugs for WNV Disease

Exploring innovative therapeutic strategies to address socially relevant diseases focuses on different molecular systems that include both natural and synthetic compounds, ranging from peptidic and oligonucleotidic molecules to chimeric structures such as nucleobase-containing peptides [182,183,184,185,186,187,188,189]. In general, biomedicine is advancing in different respects, including the development of supramolecular platforms such as metallogels for therapeutic applications [190], as well as the research into natural products in neurodegeneration [191]. Progress has also been made in understanding intracellular parasitic infections and their global burden [192], alongside precision therapeutics through bioactive compounds, omics integration, and drug repurposing strategies [193]. Moreover, convergent approaches in neurodegeneration [194], synthetic molecules for antimicrobial and antiviral applications [195,196], studies on biomacromolecular interactions and antioxidant properties of novel amino acid-based compounds [197,198,199,200,201,202,203,204,205,206] machine learning applications in neuroimaging [207], and nature-inspired covalent inhibitors for pathogens and cancer proteins [208] all illustrate the breadth of current biomedical innovation. These advances also include valorization of agri-food waste for functional products [209,210,211,212,213,214,215,216,217,218,219] as well as analytical chemistry methodologies [220,221]. Altogether, these innovations highlight the diverse directions in which biomedical science is evolving, with numerous promising strategies being under investigation for the development of an effective vaccine against WNV [165]. Recent therapeutic strategies against West Nile virus have focused on targeting both structural and non-structural proteins that are critical for viral replication and pathogenesis with different molecular strategies. In this respect, promising candidates include peptide-based inhibitors, as well as monoclonal antibodies, and small molecules designed to disrupt specific WNV-relevant protein–protein interactions.

Most current findings in this area derive from in silico predictions or wet experiments including in vitro assays, with often limited validation in in vivo models, which reveals the need for translational studies.

The already mentioned E protein, essential for virion assembly and host cell entry, has emerged as a key drug target. Its DIII domain, responsible for receptor binding, has been explored using cyclic peptides and neutralizing derivatives, some of which demonstrated not only the inhibition of the viral replication, but also the ability to cross the blood–brain barrier in specific animal models (Table 2).

Humanized monoclonal antibodies directed against DIII have also shown protective effects, even in cases of established neuroinvasion, highlighting their therapeutic potential. On the other hand, small molecules with inhibitory activities such as AP30451 and related compounds further illustrate the feasibility of blocking E protein function by interfering with RNA translation and replicon activity [5].

**Figure 4:**
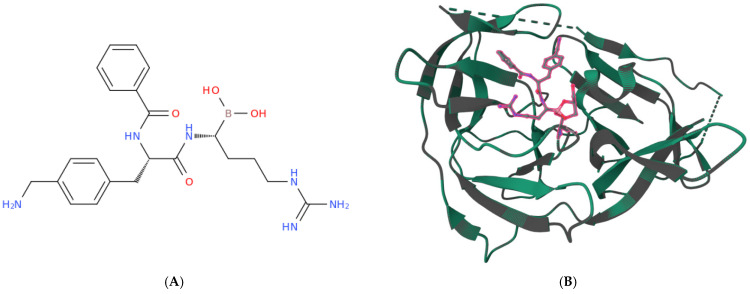
Two-dimensional structural representation of the inhibitor ((R)-1-((S)-3-(4-(aminomethyl)phenyl)-2-benzamidopropaneamido)-4-guanidinobutyl)boronic acid (**A**), and three-dimensional view (**B**) of the West Nile Virus NS2B-NS3 protease in complex with this capped dipeptide boronate inhibitor (PDB ID: 5IDK). The structure is freely available at https://www.rcsb.org/3d-view/5IDK/1, accessed on 24 November 2025.

Beyond structural proteins, non-structural proteins such as NS3 (especially in complex with NS2B) and NS5 represent another class of attractive antiviral targets. NS3 protease inhibition has been achieved with dipeptidic covalent inhibitors bearing a C-terminal boronic acid moiety, with effective inhibition of viral replication [224] (Figure 4), and by repurposed drugs like zafirlukast, synthetic inhibitors such as Cbz-Lys-Arg-(4-GuPhe)P(OPh)_2_, as well as natural product derivatives including eugenol-based triazoles, all showing potent activity in molecular docking and experimental studies [5].

Moreover, high-throughput screening has identified additional competitive inhibitors (tolcapone, tannic acid, catechol derivatives) with strong protease inhibition. Tripeptide-bound β-lactams have demonstrated dual mechanisms of NS3 inhibition, while cyclosporine has been shown to interfere with NS5-associated cyclophilin activity, though its therapeutic potential requires further in vivo validation. Further strategies include disrupting NS3-NS5 interactions with small molecules such as tyrphostin derivatives, suramin, as well as novel ZINC-database-extracted compounds, several of which exhibit broad-spectrum antiviral activity across flaviviruses and have reduced viral loads in animal models [5]. Collectively, these findings highlight a diverse pipeline of candidate potential drugs, ranging from rationally designed peptides and repurposed drugs to natural product derivatives and synthetic inhibitors. While encouraging, the majority of evidence remains preclinical when not purely computationally based, and advancing these candidates into robust in vivo validation and clinical evaluation will be crucial in order to establish effective antiviral therapies against WNV.

## 7. Conclusions

West Nile Virus constitutes a complex and persistent public health threat across the Mediterranean area and the Middle East, driven by ecological diversity, climatic variability, and migratory bird pathways that sustain viral transmission. The comparative experiences of Italy and Iran offer valuable insights into how regional differences in surveillance infrastructure, vector ecology, and public health priorities shape the trajectory of the reported WNV outbreaks that are much less severe in Iran. More in detail, Italy’s recurrent WNV epidemics, particularly in its northern regions, led to the development of robust, multi-tiered surveillance systems, integrating “One Health” approaches from the available veterinary, entomological, and human health data. Predictive modeling using different types of environmental indicators and early-warning methods has further enhanced Italy’s capacity to anticipate and to some extent contain the WNV outbreaks. On the other hand, Iran’s lower rate of clinically confirmed cases belies a widespread serological footprint, especially in southern and southwestern provinces. This silent circulation among both humans and animals highlights the importance of expanding diagnostic capacity and integrating WNV into national disease monitoring frameworks due to the potential for unpredictable future severe outbreaks, including other regions of the Middle East. Concernig WNV detection, WBE was recently employed for monitoring WNV in wastewater samples, but in our opinion, further studies comparing WBE with other surveillance methods are desirable to evaluate the efficacy of this strategy in WNV detection with respect to other standard methods. Despite extensive research efforts worldwide, neither a licensed human vaccine nor specific approved drugs for WNV currently exist. This gap should be a matter of concern for the scientific community, especially given the potential for future increases in WNV severity globally. As for vaccines, only animal-targeted WNV vaccines remain a primary valid prophylactic tool, with Italy providing a remarkable example of such successful implementation in horses. On the other hand, experimental antiviral therapies, including NS3 inhibitors, show promise in preclinical studies but have yet to yield conclusive results and, thus, effective treatments for human cases. Ultimately, the experiences of Italy and Iran show the necessity of a sustained, interdisciplinary approach rooted in the “One Health” paradigm. Coordinated efforts across human, animal, and environmental health settings, combined with advances in molecular virology, vector control, and public awareness, are all essential features if we want to mitigate the impact of this virus in a scenario of future WNV outbreaks. In this context, continued investment in drug and vaccine discovery, active surveillance, “One Health“ data sharing, and translational research will be key to developing effective prophylactic and therapeutic strategies for this enduring zoonotic challenge.

## Figures and Tables

**Table 1:** Summary of current WNV vaccine candidates, including platforms, key mechanisms, and development status.

Vaccine Platform	Specific Candidate/Strain	Key Mechanism & Features	Development Status & Key Observations	Ref.
**Virus-Like particles**	WNV VLPs (Insect/Mammalian); Mosaic nanoparticles (AP205-DIII)	Structurally mimic the virus (prM & E proteins) without genetic material; display antigens in native conformation.	Preclinical (Animal Models): Induces neutralizing antibodies; supports DIVA strategy (differentiation of infected vs. vaccinated animals) due to lack of NS proteins.	[140,141,142,143]
**mRNA vaccines**	Lipid nanoparticle (LNP) formulations	Synthetic mRNA encoding WNV antigens; facilitates endogenous protein synthesis and dual immune response (Humoral & Cellular).	Preclinical: Not yet in clinical trials for WNV; extrapolated success from Zika/Dengue models; challenges regarding cold-chain distribution (−20 °C to −70 °C).	[146,147,148,149,150]
**DNA vaccines**	Plasmid encoding prM/E (e.g., CMV/R promoter-driven)	Non-pathogenic plasmids expressing WNV glycoproteins in host cells; capable of expressing additional proteins (e.g., Capsid, NS1).	Phase I clinical trial: Safe and immunogenic; 3 doses induced neutralizing antibodies in >95% of participants (including elderly); no licensed human DNA vaccine yet.	[23,151,152,153,154]
**Inactivated (Vero-derived)**	WN-VAX (Strain NY99-35262)	Whole virus inactivated and propagated in Vero cells; uses established manufacturing technology.	Preclinical: Safe and reliable; enhanced immunogenicity observed in mice with pre-existing Japanese Encephalitis immunity.	[158,159,162]
**Live-attenuated**	WN1415 (Based on Lineage II B956)	Utilization of less virulent Lineage II strains to reduce safety concerns.	Preclinical: Low doses (55 PFU) protected ~67% of mice; higher doses provided 100% protection against lethal challenge.	[161,162]
**Chimeric vaccines**	ChimeriVax-WN02; WN/DEN4Δ30	WNV structural genes are inserted into a foreign backbone (e.g., Yellow Fever 17D or Dengue-4) with attenuating mutations.	Phase I Clinical Trial: ChimeriVax-WN02 showed 100% seroconversion in adults; reduced neurovirulence compared to parent strains.	[5,153,163,164,165,166]
**Viral vectors**	MVA-WNV (Modified Vaccinia Ankara)	Non-replicating vector expressing E-antigen; highly expressed in host cells without vector replication.	Preclinical: Complete protection against Lineage 1 & 2 strains; induces both antibody and T-cell responses; suitable for emergency vaccination.	[165,167,168]
**licensed veterinary vaccines**	Equip WNV (inactivated); Proteq WNV (recombinant); Equilis WNV (chimeric)	Various platforms (Inactivated whole virus, Canarypox vector, YF-chimera) specifically formulated for equines.	Licensed (Veterinary Use): High efficacy in horses (94–100% protection from viremia); Canarypox vector shows longer-lasting immunity (12 months); PreveNile (chimeric) was withdrawn due to adverse events.	[23,164,165,169,170,171,172,173,174,175,176]

**Table 2:** Summary of experimental therapeutic strategies against WNV, categorized by molecular target. The table includes structural (E protein) and non-structural (NS3, NS5) viral targets, listing key compound classes and their observed mechanisms of action based on in silico, in vitro, and limited in vivo studies.

Molecular Target	Compound Class/Strategy	Specific Candidates/Examples	Key Mechanism & Observations	Ref.
E Protein (Structural/DIII Domain)	Peptide-based inhibitor	Cyclic peptides; neutralizing derivatives	Inhibit receptor binding and viral replication; capable of crossing the blood–brain barrier in animal models.	[222]
E Protein (Structural/DIII Domain)	Immunotherapy	Humanized monoclonal antibodies (mAbs)	Show protective effects even during established neuroinvasion.	[5]
E Protein (Structural)	Small molecules	AP30451 and related compounds	Block E protein functions by interfering with RNA translation and replicon activity.	[223]
NS3 Protease (Non-structural)	Covalent inhibitors	Dipeptidic inhibitors with C-terminal boronic acid	Effective inhibition of viral replication.	[224]
NS3 Protease (Non-structural)	Repurposed & synthetic drugs	Zafirlukast; Cbz-Lys-Arg-(4-GuPhe)P(OPh)_2_	Potent activity shown in molecular docking and experimental studies.	[225,226]
NS3 Protease (Non-structural)	Natural product derivatives	Eugenol-based triazoles	Potent activity in experimental studies.	[227]
NS3 Protease (Non-structural)	Competitive inhibitors (HTS)	Tolcapone; Tannic acid; Catechol derivatives	Strong protease inhibition identified via high-throughput screening.	[228]
NS3 Protease (Non-structural)	Peptidomimetics	Tripeptide-bound β-lactams	Demonstrate dual mechanisms of NS3 inhibition.	[229]
NS5 Protease (Non-structural)	Host-targeting agent	Cyclosporine	Interferes with NS5-associated cyclophilin activity (requires further in vivo validation).	[230]
NS3-NS5 Interaction (PPI)	Small molecule inhibitors	Tyrphostin derivatives; Suramin	Broad-spectrum antiviral activity; reduced viral loads in animal models.	[231]

## Data Availability

No new data were created or analyzed in this study.

## Abbreviations

The following abbreviations are used in this manuscript:

AFP	Acute Flaccid Paralysis
C	Capsid Protein
DENV	Dengue Virus
DIVA	Differentiating Infected vs. Vaccinated Animals
ddRT PCR	Droplet Digital Reverse Transcription Polymerase Chain Reaction
E	Envelope Protein
ER	Endoplasmic Reticulum
G4	G quadruplex
MVA	Modified Vaccinia Ankara
NAT	Nucleic Acid Testing
NS	Nonstructural Protein
ORF	Open Reading Frame
prM	Premembrane Protein
RdRp	RNA-dependent RNA Polymerase
TGN	Trans Golgi Network
UTR	Untranslated Region
VLP	Virus-like particle
WBE	Wastewater-Based Epidemiology
WNV	West Nile Virus
WNND	West Nile Neuroinvasive Disease
WNVD	West Nile Virus Disease
ZIKV	Zika Virus
